# Case report: Leptospirosis with multi-organ failure complicated by massive upper gastrointestinal bleeding in a non-epidemic setting with successful management

**DOI:** 10.3389/fsurg.2023.1131659

**Published:** 2023-03-07

**Authors:** Mariam Thalji, Hanan Qunibi, Loai Muhtasib, Hasan Hroob, Ashraf Al-Zughayyar, Rafiq Salhab, Yousef Abu Asbeh

**Affiliations:** ^1^ Faculty of Medicine, Al-Quds University, Jerusalem, Palestine; ^2^ Medical Intensive Care Unit, Al-Ahli Hospital, Hebron, Palestine; ^3^ Genaral Surgery Department, Al-Ahli Hospital, Hebron, Palestine; ^4^ Thoracic Surgery Unit, Al-Ahli Hospital, Hebron, Palestine

**Keywords:** leptospirosis, weil’s disease, acute kidney injury, alveolar hemorrhage, multiorgan failure, upper G.I. bleeding

## Abstract

Leptospirosis is a common zoonotic disease with a wide range of clinical manifestations, specifically in tropical regions. Weil's disease is considered a severe form of leptospirosis seen in a minority of leptospirosis cases with considerable mortality. These patients typically developed the triad of acute renal injury, jaundice, and hemorrhages. Herein, we reported a case of a 28-year-old male transferred to our intensive care unit due to severe leptospirosis with diffuse alveolar hemorrhage, cholestatic jaundice, acute respiratory distress, and renal injury. The patient was successfully managed with appropriate antimicrobial treatment and other supportive management, including mechanical ventilation, vasopressor, and corticosteroid therapy. Ten days after admission, the patient unexpectedly developed uncontrollable massive upper gastrointestinal bleeding, requiring immediate surgical interventions. Splenectomy, partial gastrectomy, along with gastro-esophageal anastomosis were performed. Following a prolonged hospitalization, the patient fully recovered and was discharged home with excellent clinical outcomes. This fulminant leptospirosis case described here should assist in informing medical professionals of the clinical significance of this serious, occasionally fatal illness. Moreover, leptospirosis should be considered in any location wherever risk factors are present, not just in epidemic and tropical areas. In this case, we pointed out that serious complications of leptospirosis, such as hemorrhage, may happen despite their rarity. In such cases, adopting an integrated multidisciplinary team approach is essential to prevent complications and reduce mortality.

## Introduction

Leptospirosis is a widespread zoonotic disease caused by a pathogenic spirochete called Leptospira. This zoonosis disease is common in tropical and subtropical regions, where it significantly increases morbidity and mortality (1, 2). Leptospiral infection is transmitted to humans by infected animals, including rodents, cats, and dogs. A wide range of clinical manifestations ranges from mild subclinical illness to life-threatening conditions and even death (3). The majority of cases (90%–95%) present with an anicteric self-limited course associated with fever, headache, mild gastrointestinal symptoms, and myalgia. However, a minority of patients may manifest with icteric illness resulting in multi-organ failure, including renal, hepatic, respiratory, and circulatory failure (4, 5). The gold standard for the diagnosis is to isolate the bacteria by a blood or cerebrospinal fluid culture during the first ten days. Because of the difficulty and the long time that method requires, serologic testing such as the microscopic agglutination test (MAT) is used to diagnose most cases. There are rare reported serious leptospirosis complications comprise upper gastrointestinal bleeding (UGIB), meningoencephalitis, and necrotizing pancreatitis (6). We present a case of a 28-year-old male who developed leptospirosis complicated by massive UGIB necessitating an urgent laparotomy in which splenectomy, partial gastrectomy, along with gastro-esophageal anastomosis were performed. In this study, we will go over the complex course of the disease, as well as the entire management process that results in an excellent clinical outcome.

## Case presentation

Our patient is a 28-year-old male from Gaza who was transferred to our hospital's medical intensive care unit (ICU) because of complicated leptospirosis. His past surgical and medical history was insignificant. Ten days before admission, the patient had a rat bite in his left big toe, so he sought medical advice, where he was given a tetanus vaccine and discharged with a recommendation to follow up at the infectious clinic. Later on, the patient started complaining of nonspecific symptoms of headache, general weakness, and myalgia, for which he took analgesics. However, his symptoms worsened, and he started to notice a yellowish discoloration all over his body, associated with fever. So, he was admitted to the medical ward in Gaza, where Investigations were performed and confirmed the diagnosis of leptospirosis. Those revealed alveolar hemorrhage, cholestatic jaundice, and acute kidney injury. The patient's condition rapidly deteriorated, requiring intubation and urgent transfer to our hospital for Advanced ICU management.

**Figure 1:**
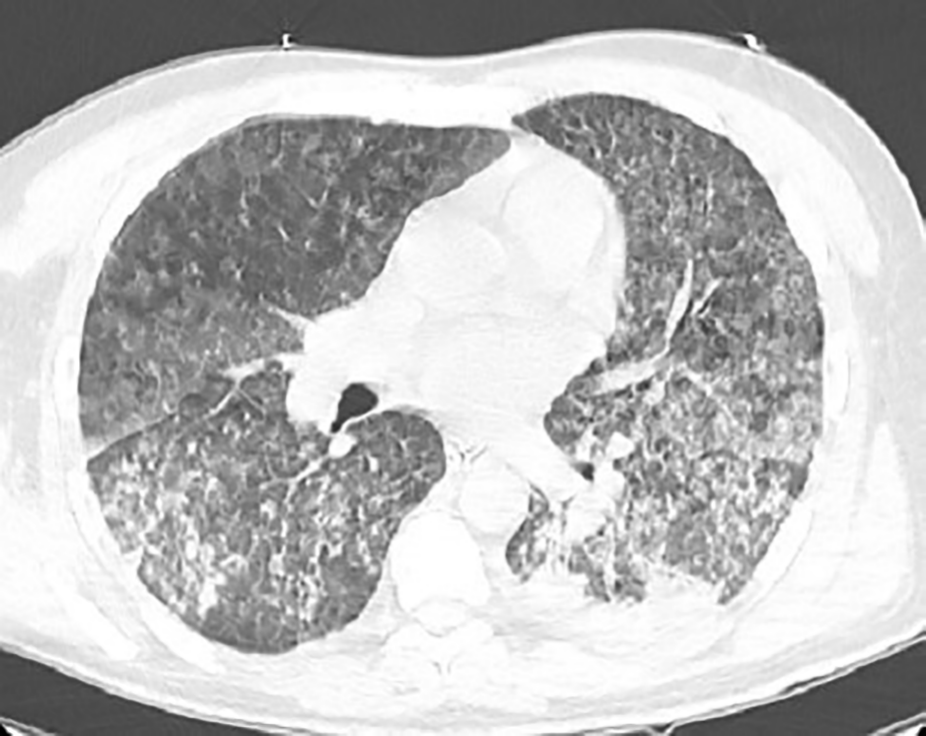
Chest CT Scan showing diffuse bilateral patchy alveolar infiltration with interlobular septal thickening and consolidation mostly seen at the posterior aspect of the lungs representing alveolar hemorrhage.

Upon arrival, the Patient was admitted to the ICU immediately. On examination, He was deeply sedated on mechanical ventilation SIMV, PEEP:10, FIO2: 100%, PS:20, RR:18). He was pale, deeply jaundiced, and ecchymosis was found on his hands and legs. Chest auscultation revealed bilateral basal crepitation. His vital signs showed a heart rate of 88 beats/min, blood pressure of 150/90 mmHg, and body temperature of 38.6°C. His blood oxygen saturation was 99% on the parameters as mentioned above. Upon admission, extensive laboratory investigations were obtained, including a full septic workup (Table 1). Arterial blood gases revealed a PH of 7.40, CO2:45.3 mmHG, and HCO3:28 mEq/L. The white blood cell count was elevated, primarily the neutrophils. He was anemic, and thrombocytopenia was present. The transaminases level was average. There was a conjugated hyperbilirubinemia. Blood urea nitrogen and serum creatinine levels were significantly increased, suggesting acute renal injury. Urine analysis showed no proteinuria. CT scan showed diffuse bilateral areas of patchy alveolar infiltration ground glass opacities, representing alveolar hemorrhage (Figure 1). The patient was started on broad-spectrum antibiotics, including vancomycin, meropenem, and doxycycline, as well as esomeprazole 40 mg orally and a Pulse dose of Methylprednisolone 1 g once daily for three days, then tapering gradually to 80 g. By the sixth day of admission, the patient developed Ventilator-associated pneumonia. Sputum culture showed pseudomonas aeruginosa sensitive for colistin.

**Table 1:** Laboratory data on admission.

Parameters	Value	Reference range
**Hematology**		
Total white blood cells count	20.5	5–10 × 10^3^/L
Neutrophils	76.8	45–65%
Lymphocytes	8.6	25–45%
Monocytes	13.2	0–6%
Eosinophils	0	0–2%
Basophils	1.3	0–1%
Hemoglobin	8.6	14–18 mg/dl
Hematocrit	25.9	40–52%
Platelets	51.3	150–400 × 103/µ
**Biochemistry and serology**		
C-reactive protein	36.1	Up to 6 mg/L
LDH	431	207–414
Creatinine	2.8	.4–1.2 mg/dl
Blood urea nitrogen	61	4.7–23.4 mg/dl
Total Serum bilirubin	20.5	Up to 1.2 mg/dl
Direct bilirubin	19.9	<.3 mg/dl
SGOT	38	0–37 U/L
SGPT	40	0–45 U/L
ALP	75	Up to 115 U/L
Albumin	2.8	3.5–5.5 g/dl
Sodium	146	132–148 mEq/L
Potassium	3.6	3.9–5.7 mEq/L
Calcium	7.1	8–5–10.4 mg/dl
**Coagulation**		
D-Dimer	1522	0–250 mg/dl
PT	13.6	
APTT	22.7	25–36
Fibrinogen	417	150–350 mg/dl
INR	1.1	

SGPT, serum glutamate pyruvate transaminase; SGOT, serum glutamic-oxaloacetic transaminase; PT, prothrombin time; APTT, activated partial thromboplastin time.

Day by day, the Patient's condition dramatically resolved. His labs showed stable blood hemostasis The creatinine, liver enzymes, bilirubin, and CRP gradually subsided. Also, his respiratory conditions improved. The patient was successfully extubated on day 10 from admission. His condition was stable, and he was transferred to the medical ward to continue antibiotics therapy. On day 18 of admission, he suddenly developed abdominal pain and severe hematemesis of a large amount of blood (about 3 liters). He became unconscious with a GCC scale of 4/15; the blood pressure was unrecordable. An Urgent resuscitative strategy for hemorrhagic shock was started, including the following: two bores IV cannula, two central lines (femoral and internal jugular), massive blood product transfusion with a total of 30 units (10 units of PRBC, 10 FFP, 10 PLTS), several liters of IV Fluid (Normal saline and ringer lactate), and calcium gluconate administration. Then Intubation was done, and adrenaline and noradrenaline were started. An urgent upper endoscopy revealed an intact oesophagus and a stomach full of blood. Laboratory results showed a Hb of 4.22 mg/dl, HCT of 15.7%, MCH of 27 PG/ML, MCV of 88 µm^3^, RBC of 1.4 × 10^6^/µl, wbc of 11 × 10^3^/L, Platelets of 50 × 103/µ, SGPT of 94 U/L, SGOT of 37 U/L, BUN of 28 mg/dl, Cre of 0.9 mg/dl, Fibrinogen of 220 mg/dl, CRP of 12.4 mg/dl, D-dimer of 910 mg/dl, PT of 13.6, INR of 1.15, APTT of 20.6, T. bilirubin of 2 mg/dl, and D. Bilirubin of 1.3 mg/dl.

**Figure 2:**
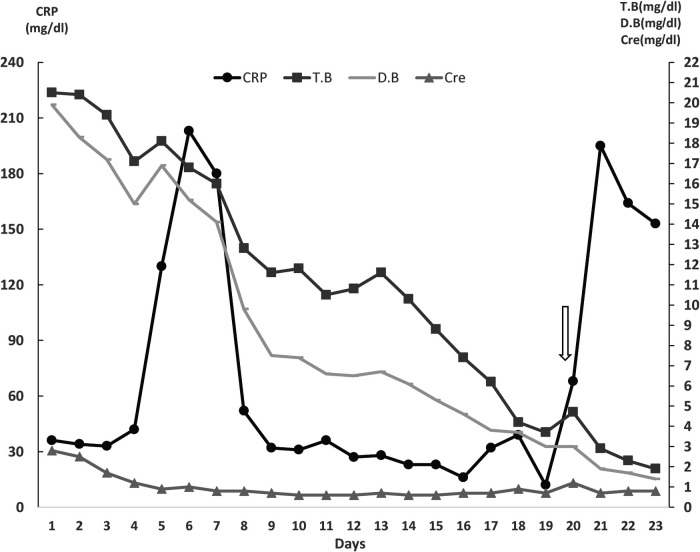
Clinical course of the patient during 23 days of hospitalization in the ICU. (Arrow represents the day in which Upper Gastrointestinal bleeding happened). CRP, C-reactive protein; CRE, creatinine; T.bil, total Bilirubin; D.bil, Direct Bilirubin.

The patient underwent urgent exploratory laparotomy. Open stomach anterior wall and gastric lumen packing were done. The upper half of the mucosa of the stomach oozed fresh blood, and a Splenic artery aneurysm eroded into the gastroesophageal junction. So left gastric artery was ligated, Splenectomy and proximal partial gastrectomy with distal esophagectomy were performed. Intraoperatively, the Patient receives 8 PRPC, 10 FFP, 10 PLT,2500-unit prothrombin complex concentration, and 8,000 CC IV fluid. Afterward, the Patient was readmitted to the ICU, fully sedated and intubated. The next day, a second look procedure was done involving esophagogastric anastomosis and anterior continues repair of the wall. A jejunostomy feeding tube along with abdominal drains was applied. Figure 2 Showed the Patient's clinical course during the first 23 days of hospitalization.

**Figure 3:**
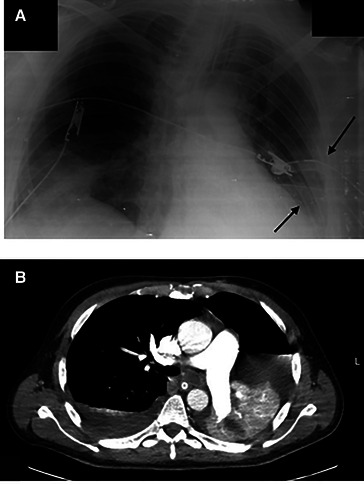
(**A**) Portable x-ray demonstrating two chest tubes inserted at the left side (arrows). (**B**) CT scan showing Collapsed left lung, causing ipsilateral deviation of the mediastinum and trachea, mild to moderate left sided pneumothorax, and mild bilateral pleural effusion. Focal area of consolidation seen at the right lower lobe with surrounding ground glass opacities.

5–6 days later, the Patient complained of shortness of breath and chest pain. A physical exam revealed tachypnea, desaturation, crackles on the right side, and decreased breath sounds on the left side. A portable chest x-ray showed pleural effusion and left-sided pneumothorax. CT scan findings were: Collapsed left lung due to complete obstruction of the left main bronchus. Mild to moderate left-sided pneumothorax, mild bilateral pleural effusion. A focal consolidation area is seen at the right lower lobe with surrounding ground glass opacities. Two Chest tubes were inserted at the left side, and the Patient's condition improved (Figure 3). The Patient underwent a bronchoscopy, which showed a left main bronchus occlusion. Multiple bronchoscopies were performed by which large amounts of secretions were removed.

**Figure 4:**
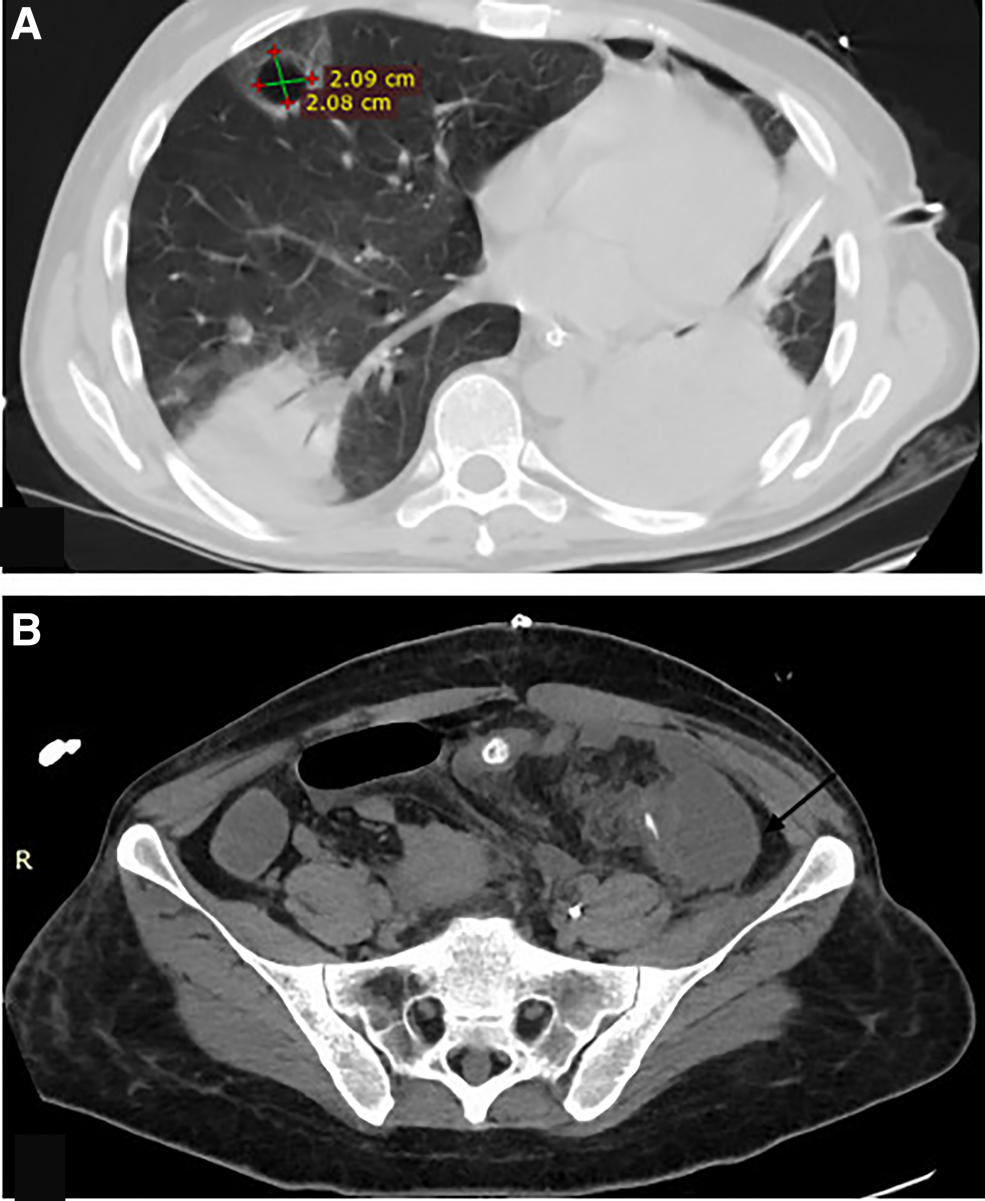
(**A**) Chest -Ct scan revealing a right upper lobe cavitary lesion surrounded by ground glass opacities, measuring about 2 × 2 cm.(**B**) Abdomen CT scan intraabdominal fluid collections at the left paracolic getter measuring about 4.5 × 4.5 × 10 cm (arrow) with adjacent smaller one measuring about 3 × 4.5 × 5 cm.

The Patient developed an unresolved fever. Full septic work was done, and Antibiotics were upgraded. Chest and abdomen Ct scan with contrast revealed a right upper lobe cavitary lesion surrounded by ground glass opacities. Multiple intraabdominal fluid collections were noted (Figure 4). Ultrasound Guidance drainage was done. Fluid culture revealed stenotrophomonas bacteria sensitive for colistin, levofloxacin, and resprim. The patient was given Colistin and resprim.

Day by day, the Patient showed gradual resolution of his status and was successfully extubated. Extensive supportive strategies were followed for recovery from critical illness, including exercise rehabilitation and physiotherapy. He was transferred to the medical world and discharged home. On follow up the patient was in a good general condition.

## Discussion

We presented a severe case of leptospirosis in a healthy 28-year-old male. In the early phase of the illness, the patient displayed fever, headache, and myalgia but still had no other organ involvement. However, his condition worsened later because of multiple organ failure and massive upper gastrointestinal bleeding (UGIB), necessitating immediate surgical management. Leptospirosis is a tropical zoonotic disease caused by L.interrogans, a flagellated, motile, and spiral-shaped bacteria. Humans contract the disease directly or indirectly by contacting the infected animal's urine or contaminated soil or water (7, 8). Rat bite is an unusual mode of transmission that results in a direct infection, as in our patient. Even though it's a rare mode of transmission, some cases have been reported (9, 10).

Leptospirosis can affect several organs, resulting in a variable clinical picture that's related to the involved organs. Renal manifestations include uremia, oliguria, or hematuria. Moreover, interstitial nephritis and significant tubular damage may develop in severe cases. Considering the pulmonary involvement, studies showed that the mortality rate increased from 4.8% to 11% in leptospirosis patients with pulmonary involvement. These symptoms range from mild chest pain, dyspnea, and cough to devastating conditions such as pulmonary haemorrhage or acute respiratory distress syndrome (8, 11, 12). A hemorrhagic pulmonary syndrome is considered a leading cause of death in leptospirosis, and most affected cases require mechanical ventilation. Further, it has been found that severe pulmonary hemorrhagic syndrome patients have a 52% mortality risk when hospitalized in the ICU (13). Hepatic impairment is generally minimal and reversible. Liver injury can be detected in severe leptospirosis as conjugated bilirubin levels rise above 80 mg/dl, accompanied by mild increases in transaminases that infrequently exceed 200 U/L. Thrombocytopenia has been reported with severe leptospirosis. However, both thrombocytopenia and hemorrhagic pathogenesis in leptospirosis are poorly understood (8).

Weil's disease is a serious form of leptospirosis, with a mortality rate of more than 50% (14). It mostly happens during the second phase, but it may happen at any point during the disease. Patients typically had fever jaundice, hepato- renal failure, and pulmonary or gastrointestinal haemorrhage with variable severity. UGIB related to leptospirosis is mentioned in 10.3% of cases, and about 3.4% with active bleeding that need endoscopic clipping (15). One explanation for the pathophysiology of the reported haemostatic diathesis suggests that leptospira acts directly on endothelial cells (16).

As in our patient's condition, he had Jaundice, acute kidney injury, diffuse alveolar pulmonary haemorrhage, and ARDS. He also developed massive UGIB. that was impossible to identify the active source of bleeding by gastroscopy. The patient continued to have hemodynamic instability and persistent bleeding, so a multidisciplinary team discussion was done, including a thoracic surgeon, vascular surgeon, ICU specialists and Gastroenterologist. The plan was to go for an urgent laparotomy.

Mild leptospirosis cases typically resolve without antibiotics; nevertheless, some patients experience complications that result in considerable morbidity and mortality. Antimicrobial agents are the mainstay in the treatment of such cases since it helps to prevent or delay the development of the immune phase, thus reducing complications. Doxycycline is an effective therapy for mild cases, especially in low-resource settings. Severe cases need a Combined antibiotic with Parenteral penicillin, doxycycline, and third-generation cephalosporins rather than one agent alone (17, 18). Furthermore, leptospirosis patients with multiorgan failure are usually admitted to ICU because of their critical illness. This makes them at a high risk of getting hospitalized infections such as pseudomonas aureginosa, as in our case.

According to previous reports, severe cases may benefit from corticosteroid therapy, considering the vasculitis nature of severe leptospirosis, particularly in instances with thrombocytopenia and pulmonary involvement (19). In our patient, a 3-day 1 g per day methylprednisolone was started 24 h following ICU admission with gradual tapering of the dose. However, more studies are needed to support the corticosteroid use.

## Conclusion

The implications of this study are notable for many reasons. Leptospirosis continues to be a cause of infection in developing countries. Early recognition is crucial as early antibiotic administration can decrease the severity and duration of the disease and lead to excellent outcomes. This case is reported to remind physicians that rare serious complications such as GI bleeding may happen. A multidisciplinary team discussion is recommended for better patient survival.

## Data Availability

The original contributions presented in the study are included in the article/Supplementary Material, further inquiries can be directed to the corresponding author/s.
